# Insulin receptor knockdown blocks filarial parasite development and alters egg production in the southern house mosquito, *Culex quinquefasciatus*

**DOI:** 10.1371/journal.pntd.0006413

**Published:** 2018-04-12

**Authors:** Andrew Bradley Nuss, Mark R. Brown, Upadhyayula Suryanarayana Murty, Monika Gulia-Nuss

**Affiliations:** 1 Department of Agriculture, Nutrition, and Veterinary Sciences, University of Nevada, Reno, Nevada, United States of America; 2 Department of Biochemistry and Molecular Biology, University of Nevada, Reno, Nevada, United States of America; 3 Department of Entomology, University of Georgia, Athens, Georgia, United States of America; 4 Department of Biology, Indian Institute of Chemical Technology, Hyderabad, India; University of Cincinnati, UNITED STATES

## Abstract

Lymphatic filariasis, commonly known as elephantiasis, is a painful and profoundly disfiguring disease. *Wuchreria bancrofti* (*Wb*) is responsible for >90% of infections and the remainder are caused by *Brugia spp*. Mosquitoes of the genera *Culex* (in urban and semi-urban areas), *Anopheles* (in rural areas of Africa and elsewhere), and *Aedes* (in Pacific islands) are the major vectors of *W*. *bancrofti*. A preventive chemotherapy called mass drug administration (MDA), including albendazole with ivermectin or diethylcarbamazine citrate (DEC) is used in endemic areas. Vector control strategies such as residual insecticide spraying and long-lasting insecticidal nets are supplemental to the core strategy of MDA to enhance elimination efforts. However, increasing insecticide resistance in mosquitoes and drug resistance in parasite limit the effectiveness of existing interventions, and new measures are needed for mosquito population control and disruption of mosquito-parasite interactions to reduce transmission. Mosquito insulin signaling regulates nutrient metabolism and has been implicated in reduced prevalence and intensity of malaria parasite, *Plasmodium falciparum*, infection in mosquitoes. Currently no data are available to assess how insulin signaling in mosquitoes affects the development of multi-cellular parasites, such as filarial nematodes. Here, we show that insulin receptor knockdown in blood fed *C*. *quinquefasciatus*, the major vector of *Wb* in India, completely blocks the development of filarial nematode parasite to the infective L3 stage, and results in decreased ecdysteroid production and trypsin activity leading to fewer mosquito eggs. These data indicate that a functional mosquito insulin receptor (IR) is necessary for filarial parasite development and mosquito reproduction. Therefore, insulin signaling may represent a new target for the development of vector control or parasite blocking strategies.

## Author summary

Lymphatic filariasis (LF) is caused by infection with nematodes of the family Filarioidea. 90% of infections are caused by *Wuchereria bancrofti* and the remainder by *Brugia spp*. In endemic countries, LF has a major social and economic impact with an estimated annual loss of $1 billion. Filarial infection can cause a variety of clinical manifestations, including lymphoedema of the limbs, genital disease (hydrocele, and swelling of the scrotum and penis) and recurrent acute attacks, which are extremely painful and are accompanied by fever. As one of the leading causes of global disability, LF accounts for at least 2.8 million disability-adjusted life year (DALY). Mass drug administration (MDA) is used prophylactically on the community level where the infection is present to decrease disease transmission. These drugs have limited effect on adult parasites but effectively reduce microfilariae in the bloodstream and prevent the spread of microfilaria to mosquitoes. Use of mosquito population control strategies is supplemental to the core strategy of MDA. However, increasing insecticide resistance in mosquitoes and drug resistant nematode parasites are complicating elimination efforts and emphasizes the need for novel interventions for vector control and parasite transmission. Insulin signaling is a highly conserved signaling pathway that regulates growth and nutrient homeostasis in animals. Our previous work in *Aedes aegypti* mosquitoes showed additional roles of insulin receptor signaling in blood digestion and reproduction. The present data strongly supports our previous findings in a different mosquito species and further explores the role of mosquito insulin receptor in the development of the filarial nematode to the infective stage. This information is pertinent to ongoing efforts to control and eradicate filariasis because insulin signaling may represent a new target for the development of vector control or transmission blocking strategies.

## Introduction

Lymphatic filariasis (LF) is one of the neglected tropical diseases and a major cause of permanent and long-term disability worldwide. The disease is caused by three species of nematode worms (filariae)–*Wuchereria bancrofti* (Wb), *Brugia malayi*, and *Brugia timori*, which are transmitted by several mosquito species within the genera *Culex*, *Anopheles*, *Aedes*, and *Mansonia*. Humans are the exclusive host of infection with *W*. *bancrofti*, complicating efforts to study this parasite in the laboratory. The principal vectors in endemic areas are dictated by the habitat and geographical range of competent mosquito species. For instance, the southern house mosquito, *Culex quinquefasciatus*, is the major vector of *Wb* in India. India constitutes approximately 45% of the world’s LF burden where ~550 million people are at risk of infection, with 59 million infected and of which 19.6 million exhibit filariasis symptoms [1–2]. Worldwide, an estimated 120 million people across 55 countries are infected with *Wb*, leading to a loss of 5.9 million disability-adjusted life-years [3].

The availability of safe, single-dose, drug treatment regimens capable of suppressing microfilariae to very low levels has resulted in targeting of this mosquito-borne disease for global elimination [4]. The Global Program to Eliminate Lymphatic Filariasis was launched in 2000 with the principal objective of breaking cycles of transmission of *Wb* and *Brugia spp*. through annual mass drug administrations (MDAs) to entire at-risk populations. In India, a MDA program has been in effect since 2004 in districts where filariasis is endemic [2]. However, emerging challenges to this approach have raised questions regarding the effectiveness of using MDA alone to eliminate LF without the inclusion of supplementary vector control. The World Health Organization is now considering vector control as a critical component of LF reduction [5]. However, current strategies of vector control have their own limitations and challenges, such as increasing insecticide resistance among vectors, and new measures are needed for mosquito population control and disruption of mosquito-filarial worm interactions to reduce transmission [6].

**Fig 1:**
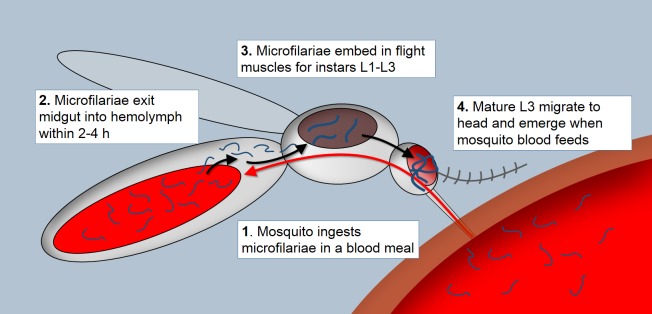
Schematic diagram of *Wuchereria bancrofti* development within a female mosquito host.

As part of the transmission cycle, mosquitoes first ingest microfilariae (mf) in blood taken from infected human hosts. The ingested mf penetrate the midgut epithelium and move into the hemolymph within 1–2 h post blood meal where they molt to first instar larvae and invade the flight muscles. After two molts, the third-stage infective larvae (L3) exit the flight muscles and lodge in the head cavity, from which they escape when the mosquito takes another blood meal [7] (Fig 1). Biological transmission of filarial worms is classified as cyclodevelopmental because the parasite undergoes development within the vector to become infective to the vertebrate host, but does not multiply [8–9]. Their successful transmission to another human host is entirely dependent on the ability of the mosquito vector to acquire and mobilize nutrients that sustain parasite growth and enable tissue repair and survival for the 10–14 days required for filarial nematode development [8]. Previous studies showed that mosquito flight muscle cells become devoid of glycogen granules following infection with *Brugia spp*. parasites [10–11], suggesting carbohydrate metabolism is essential for nematode development.

Carbohydrate metabolism and reproduction in female mosquitoes are regulated by insulin signaling. Mosquitoes, like most other insects, encode multiple insulin like peptides (ILPs) but only one receptor tyrosine kinase homolog of the insulin receptor (IR) [12]. Blood and sugar feeding by the yellow fever mosquito, *Aedes aegypti*, triggers medial neurosecretory cells in the brain to release ILPs into the hemolymph that bind to the IR and activate the insulin signaling pathway in different tissues [13–15]. Female *Ae*. *aegypti* decapitated within 1 h post sugar meal, to prevent release of endogenous ILPs, fail to store circulating carbohydrates, whereas a single dose of ILP3 directs circulating carbohydrates into glycogen and lipid stores to the same levels measured in intact females [13]. In blood fed *Ae*. *aegypti* mosquitoes, IR knockdown negatively impacts fecundity [15], immune response [16], and delays blood digestion in the midgut [15].

Some mosquito-borne pathogens are negatively affected by altered insulin signaling in mosquitoes. For example, IR knockdown in the African malaria mosquito, *Anopheles gambiae*, resulted in lower *Plasmodium berghei* loads [17]. Knockdown of two ILPs, *ILP3* and *ILP4*, in the Asian malaria mosquito, *An*. *stephensi*, also resulted in reduced prevalence and intensity of *P*. *falciparum* infection [18]. In transgenic *An*. *stephensi*, overexpression of Akt, a nexus of insulin signaling, in the midgut of heterozygous mosquitoes resulted in 60–99% reduction in the numbers of mosquitoes infected with *P*. *falciparum*, and parasite infection was completely blocked in homozygous transgenic mosquitoes [19]. Currently no data are available to assess how insulin signaling in mosquitoes affects the development of multi-cellular parasites, such as filarial nematodes.

We hypothesized that insulin signaling maintains nutrient homeostasis in blood fed, *Wb*-infected *C*. *quinquefasciatus*, which aids egg and filarial nematode development, and that mosquito *IR* (*CqIR*) knockdown would disrupt these processes. Therefore, the aim of this study was to determine the effect of *CqIR* knockdown on the development of *Wb* and the reproductive physiology of *C*. *quinquefasciatus*.

## Materials and methods

### Ethics statement for mosquito feeding on *Wb*-infected human hosts

This study was carried out in strict accordance with the recommendation of the Government of India institutional review board (IRB) for blood collection from patients. Venous blood was collected from consenting infected human volunteers (adults over 18 years, both sexes) in the Karimnagar district, Andhra Pradesh. The study was explained to the volunteers and oral consent was obtained. Written consent could not be obtained because the night clinic was in a rural area and most adults were uneducated and were unable to read and write. The IRB approved the use of oral consent, and the oral consent was documented by the health department authorities. The document contained name of the volunteer, father or husband’s name (head of the household), date of birth, signs and symptoms of elephantiasis, prior MDA obtained, and any sickness four weeks prior to the night clinic. The information was read and signed by the village head. A night clinic was established so blood could be collected between 8–11 pm local time (when the parasite is active in blood circulation) into K_2_EDTA vacutainers (BD Vacutainer, Gurgaon, India). Blood samples were kept on ice, and the number of viable mf was counted the next morning with Jaswant Singh and Bhattacharjee stain II (JSB II) [20].

### Mosquitoes

Pupae of *C*. *quinquefasciatus* were field collected from a water retention pond in Hyderabad, India and identified to species [21]. Adults reared from these pupae were blood fed on the forearm of a consenting adult for egg production. Hatched larvae were fed ground Tetramin flakes. All stages were maintained at 12 h:12 h day-light cycle at 28°C and 80% relative humidity. Adults were fed on 10% sucrose solution *ad libitum*. Mosquitoes were raised for two generations in the laboratory before conducting experiments.

### RNA interference

Double-stranded RNA (dsRNA) for *C*. *quinquefasciatus IR* (CPIJ005469) and a non-specific gene not present in *C*. *quinquefasciatus*, enhanced green florescence protein (*eGFP*), was synthesized as described previously [15]. The *CqIR* PCR product generated with T7CqIRFwd-5’TAATACGACTCACTATAGGGAGCAGTTCAACACGAACCACGTC3’ and T7CqIRRev- 5’TAATACGACTCACTATAGGGCGTCATGCCAAAGTCGCCGAT3’ was used as a template for *in vitro* transcription, following the manufacturers’ protocol (T7 Mega Script kit, Ambion). 50 females (24 h old) were injected with either *CqIR* dsRNA or control *eGFP* dsRNA (2 μg/ 0.5 μl). Mosquitoes were kept on 10% sucrose solution for 5 days for recovery and on day 6 were fed mf-infected or uninfected blood through glass-jacketed artificial membrane feeders attached to a circulating water bath (37°C). Feedings were carried out in the evening after sunset. The feeders were covered with a black blanket to simulate night. Hemotek feeding membrane (Hemotek) was kept on human ankles under socks for a minimum of 2 hrs to absorb human odor and the authors exhaled into the cages periodically to provide CO_2_ stimulation.

Total RNA was extracted from head, midgut, ovaries, and abdomen at 1, 7, and 10 days post blood meal using Trizol reagent (Invitrogen) following the manufacturer’s protocol, DNase (Invitrogen) treated, and re-purified with Trizol reagent. DNase-treated total RNA (1 μg) was used for cDNA synthesis (iScript cDNA synthesis kit, BioRad). All cDNA samples were diluted 10-fold and used as template for RT-PCR with primers for CqIRFwd- 5’GAACCACGTCGTCCGACTGCTCG3’ and CqIRRev- 5’TCGTCATGCCAAAGTCGCCGAT 3′. RT-PCR was carried out under the following conditions: initial denaturation at 95° for 5 min, denaturation at 95° for 20 sec, annealing at 56° for 30 sec and extension at 72° for 30 sec, final extension at 72° for 10 min. Primers for *Ae*. *aegypti* actin were used as a control [15].

### Parasite development assay

Five mosquitoes per treatment group (*CqIR* and *eGFP* dsRNA injected) were dissected 2–4 h post blood meal (PBM) to confirm the infectivity by presence of mf either in the gut lumen or in the hemolymph by microscope. Thirteen days PBM, an additional 6–10 mosquitoes per treatment were dissected to count mature L3 in thorax and head cavity. Experiments were repeated twice with different cohorts of mosquitoes fed infected blood. Data were analyzed by ANOVA with GraphPad software (GraphPad Software Inc. La Jolla, CA).

### Mosquito physiology bioassays

To examine fertility, five blood fed females per treatment per cohort were kept individually in small cages lined with moist paper towel. The number of eggs deposited by each female was counted 5 days PBM. To examine blood digestion, trypsin-like activity was measured in midguts dissected from a minimum of three females per treatment (uninfected +*eGFP* dsRNA injected, infected+ *eGFP* dsRNA injected, infected + *CqIR* dsRNA injected) at 24 and 48 h PBM as described previously [15]. Each treatment was replicated twice. Briefly, each midgut was transferred to 100 μl of 20 mM Tris (pH 8.0 with 20 mM CaCl_2_), sonicated, and then centrifuged (14,000 x *g* for 2 min). Midgut supernatants were frozen (−80°C), and aliquots (0.05 equivalent) were added to 100 μl of 4 mM Nα Benzoyl-L- Arginine-p-Nitroanilide (BApNA) for 10 min followed by measurement of absorbance at 405 nm using a BioTek plate reader. Activity was quantified based on trypsin standards (bovine pancreas, Sigma T1426).

Ovaries collected from the aforementioned females were used for the *in vitro* assay of ecdysteroid production. Ovaries (three pairs / 60 μl in a 1.7 ml polypropylene tube cap) in duplicate (3 pairs per cap; 2 caps) were dissected from different treatment groups (uninfected + *eGFP* dsRNA injected; infected + *eGFP* dsRNA injected; infected + *CqIR* dsRNA injected) at 24 and 48 h PBM and incubated in buffered saline [13] for 6 h at 27°C. Media (50 μl per cap) were collected and stored at −80°C until assayed for ecdysteroid content by radioimmunoassay (RIA), as described in Brown et al. [13]. Experiments were replicated twice (each with two sets of three ovaries) using different female cohorts. Mortality was also recorded daily throughout the experiment. Data were combined from both biological replicates and analyzed by ANOVA with GraphPad software.

### Accession numbers

Culex quinquefasciatus Insulin Receptor, CPIJ005469

## Results

### Mosquito IR knockdown blocks *W*. *bancrofti* larval development

**Fig 2:**
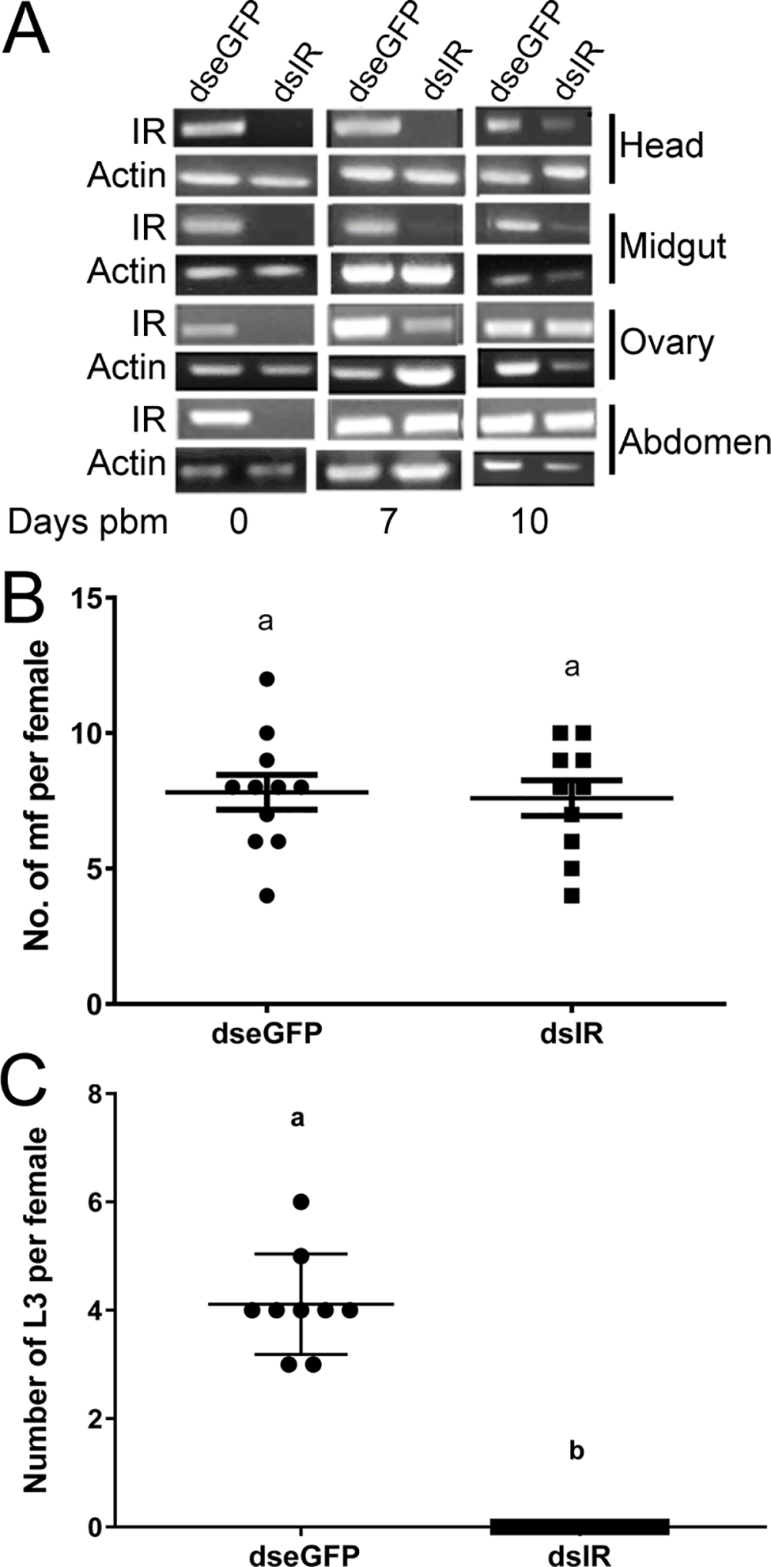
Effect of insulin receptor RNA interference on *W*. *bancrofti* development in *C*. *quinquefasciatus*. A) RT-PCR analysis of *insulin receptor* expression in tissues of control (dseGFP) and CqIR (dsIR) dsRNA injected *C*. *quinquefasciatus* before a blood meal (0), 7, and 10 days post blood meal (PBM). RT-PCR images are representative of three replicates from different biological cohorts. B) Number of microfilariae in hemolymph of control (dseGFP) and CqIR (dsIR) dsRNA injected females 2–4 h PBM. C) Number of infective L3 larvae in thorax and head of control (dseGFP) and CqIR (dsIR) dsRNA injected females 13 days PBM.

Reduced expression of the *CqIR* in *C*. *quinquefasciatus* tissues after dsRNA injection was confirmed by RT-PCR (Fig 2A). *CqIR* transcription was silenced for up to 10 days PBM (Fig 2A) in head and midgut. In ovaries and abdomen (including the fat body), *CqIR* transcript levels recovered 7 days PBM. *CqIR* knockdown had no impact on mf ingestion and movement from midgut to hemolymph of the mosquitoes (Fig 2B). By 4 h PBM, most mf migrated to hemolymph from the midgut and were visibly motile in both control *eGFP* and *CqIR* dsRNA injected females. At day 13, *eGFP* control mosquitoes had L3 in the head cavity with the exception of one L3, which was still in the thorax. In *CqIR* knockdown mosquitoes, there were no L2 or infective L3 stage either in the thorax or in the head cavity (Fig 2C). No melanized larvae were observed in the hemolymph or in the flight muscles of the thorax in the mosquitoes of either group.

### Mosquito IR knockdown and parasite infection reduces midgut trypsin activity

Given the collecting and rearing circumstances, limited numbers of *C*. *quinquefasciatus* females were available. In addition, only half or fewer of the *CqIR* dsRNA injected females were attracted to the artificial blood feeders. For these reasons, we fed only mf-infected blood to the *CqIR* dsRNA injected females to maximize the sample size for the following tissue assays.

**Fig 3:**
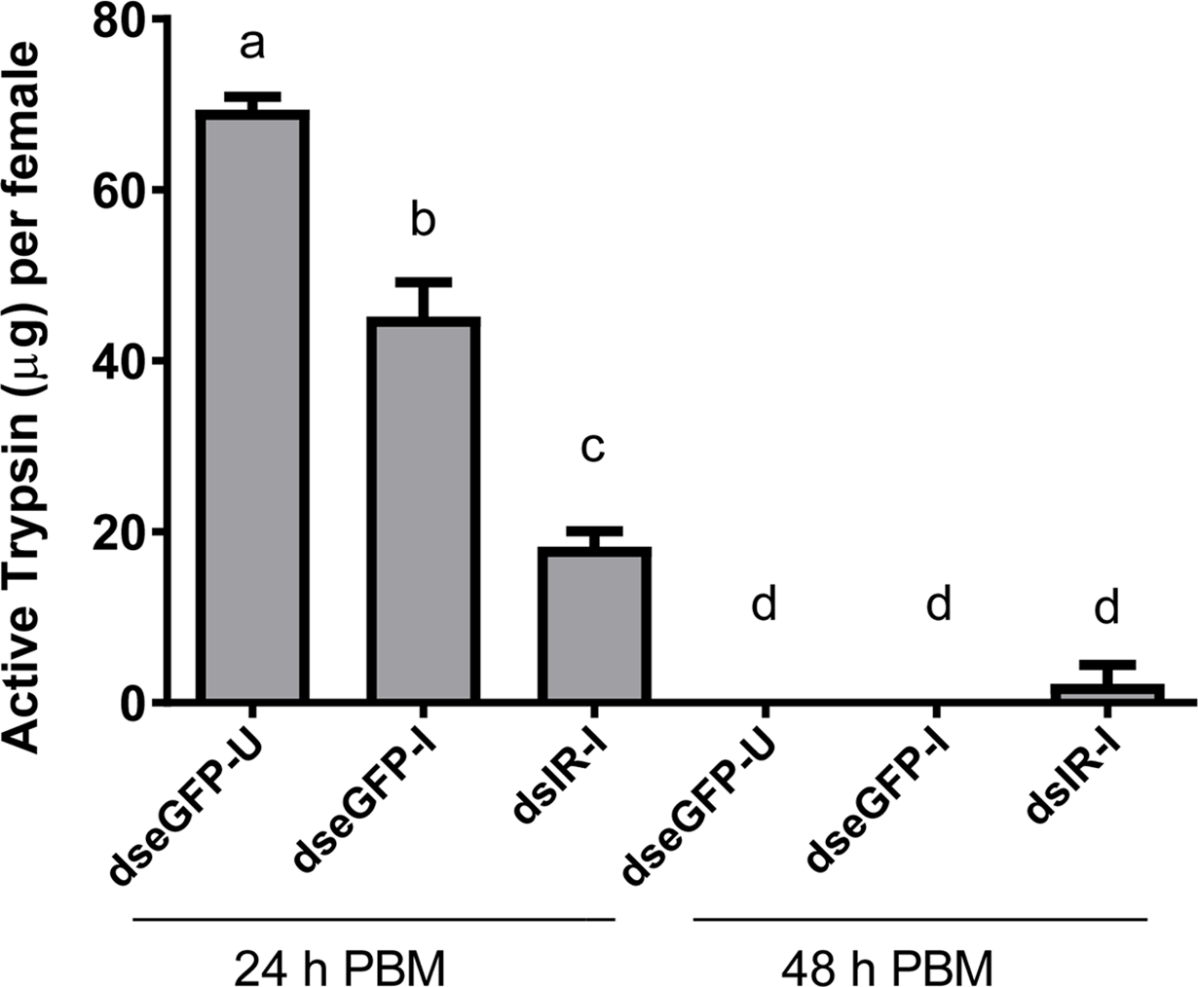
Effect of IR knockdown and nematode infection on trypsin activity. *C*. *quinquefasciatus* females were injected with either control (dseGFP) or CqIR dsRNA (dsIR) and 5 days post-injection were allowed to feed on uninfected or infected blood through artificial membrane feeders. At 24 and 48h PBM, midguts were collected for the trypsin assay with BApNA as a substrate. Treatments: dseGFP-injected and un-infected blood fed (dseGFP-U); dseGFP-injected and mf infected blood fed (dseGFP-I); dsIR- injected and mf infected blood fed (dsIR-I). Different letters above a given bar indicate means that significantly differ (F_5, 13_ = 138.4; P<0.0001). One-way ANOVA and Tukey's multiple comparison tests were used.

Trypsin enzyme activity was previously characterized in the midgut of blood fed *C*. *quinquefasciatus* [22]. It rises at ~4h PBM, peaks around 30–36 h PBM and then decreases until 72 h PBM. We assayed midguts at 24h PBM and found that *eGFP* control and un-infected mosquitoes had the highest level of trypsin activity. Parasite infection alone significantly decreased trypsin activity. Infected *CqIR* knockdown mosquitoes had the lowest trypsin activity. At 48 h PBM, trypsin activity was virtually undetectable in all three treatment groups (Fig 3).

### CqIR knockdown, not parasite infection, disrupts ecdysteroid production by ovaries

**Fig 4:**
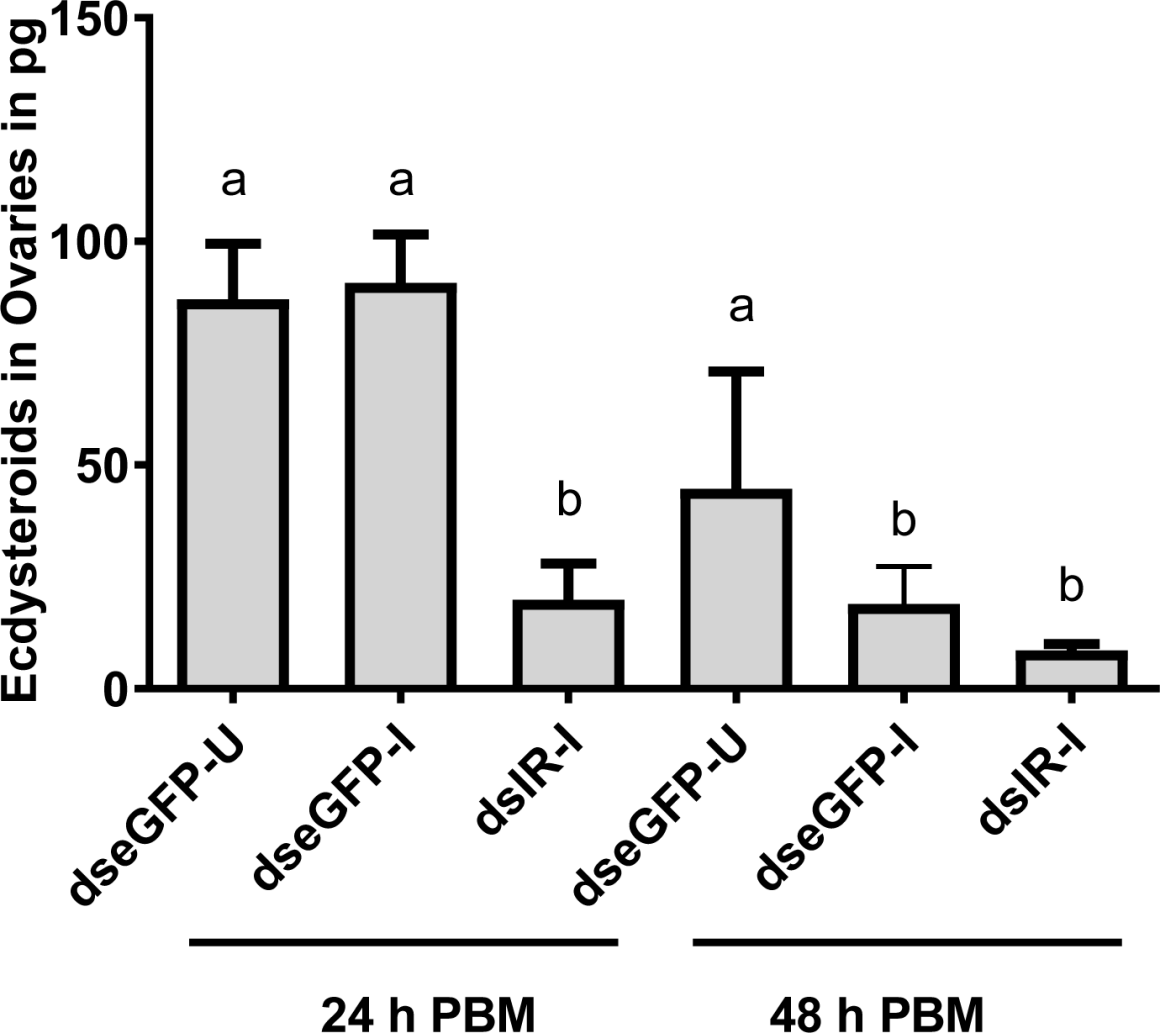
Effect of IR knockdown and nematode infection on ECD production by ovaries. *C*. *quinquefasciatus* females were injected with either control (dseGFP) or CqIR (dsIR) dsRNA and 5 days post-injection were allowed to feed on uninfected or mf infected blood. At 24 and 48h PBM, ovaries were dissected for incubation (6 h) then media was collected for quantification with the ecdysteroid radioimmunoassay. Data from each set with mean and SEM is plotted. Different letters above a given bar indicate a mean that significantly differs (F _5, 12_ = 7.078, P<0.001). One-way ANOVA with multiple comparison and Tukey's multiple comparison tests were used.

For blood feeding *C*. *pipiens* mosquitoes, ovary ecdysteroid production and hemolymph titers increase within a few hours PBM, peak 24–36 h later, and then decline rapidly [23]. We dissected and incubated ovaries *in vitro* from blood fed *eGFP* control and *CqIR* knockdown females to determine ecdysteroid production at 24 and 48h PBM. *Wb* infection did not affect ecdysteroid production by ovaries as comparable amounts were produced by ovaries from *eGFP* control females given an uninfected blood meal (Fig 4). However, ovaries from *CqIR* dsRNA injected females produced a significantly lower amount of ecdysteroids. Ecdysteroid production by ovaries taken from *eGFP* control (uninfected) females at 48 h PBM was variable–the amount in one sample was equal to that produced by ovaries at 24 h PBM, but the others were significantly lower.

### CqIR knockdown and parasite infection decrease mosquito fecundity

**Fig 5:**
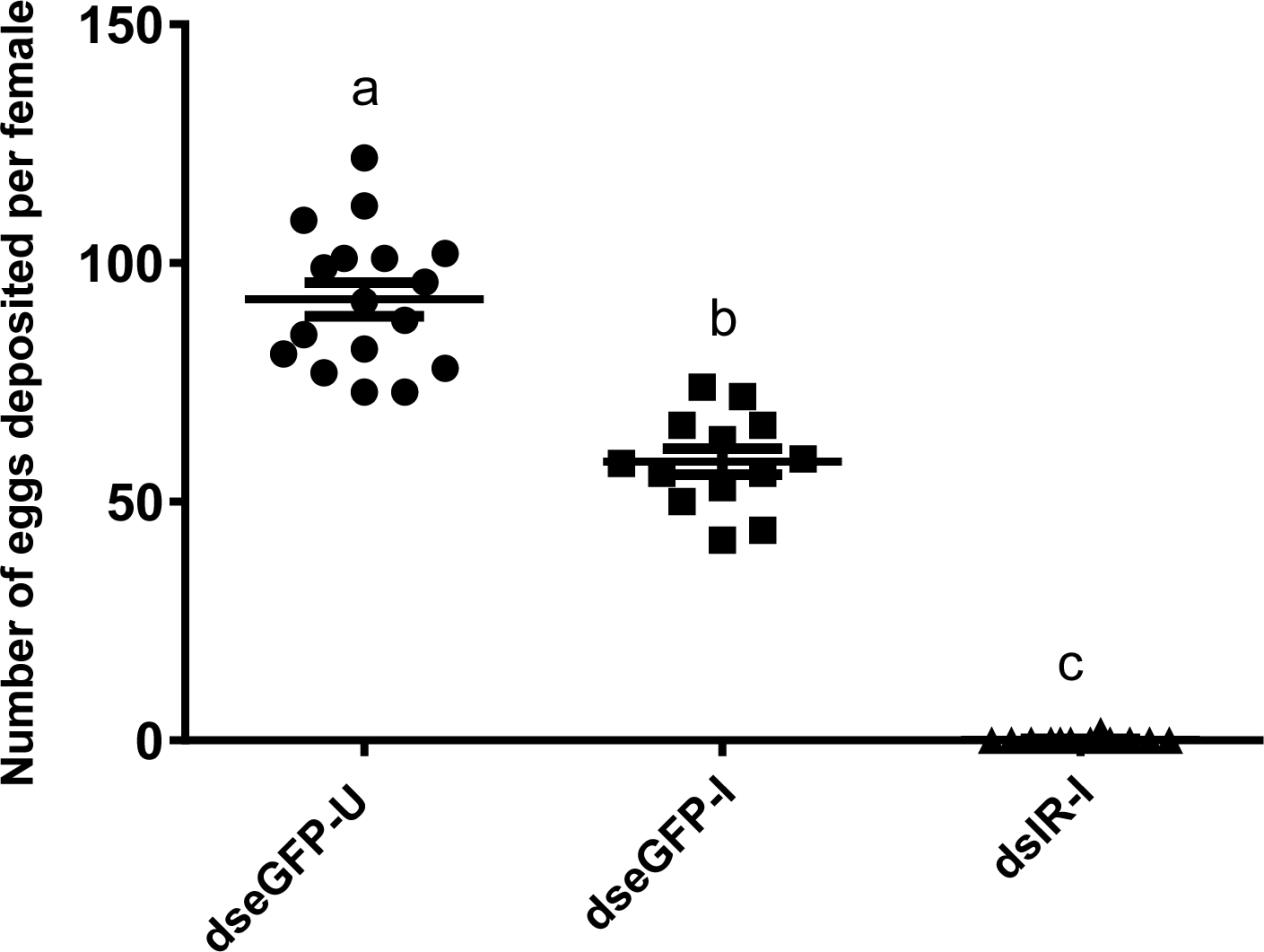
Effect of IR knockdown and nematode development on mosquito fecundity. *C*. *quinquefasciatus* females were injected with either control (dseGFP) or CqIR (dsIR) dsRNA. 5 days post injection eGFP dsRNA injected mosquitoes were fed on uninfected blood (dseGFP-U) and mf-infected blood (dseGFP-I), and dsIR injected females were fed only on infected blood-fed (dsIR-I). Total number of eggs deposited by each set of mosquitoes was counted 7 days PBM. This experiment was replicated twice with different cohorts of mosquitoes and infected blood. Data from each set with mean and SEM is plotted. Different letters above a given bar indicate means that significantly differ (F _2, 39_ = 260.1; P<0.0001). One-way ANOVA and Tukey's multiple comparisons test were used.

Control, uninfected *eGFP* females deposited an average of 92 eggs per female within 96 h PBM, whereas those infected with *Wb* produced an average of 58 eggs per female; 37% fewer. No eggs were deposited by *CqIR* dsRNA injected mf-infected females (Fig 5).

## Discussion

In this study, we provide insights into the role of insulin signaling in the reproductive and digestive physiology of *C*. *quinquefasciatus*, and how this influences the development of *Wb*, an important pathogen transmitted by this species. In *eGFP* control and *CqIR* dsRNA injected females given an infected blood meal, the mf moved out of the midgut and into the hemocoel within 2 h. Previous studies also suggested mf moved into the hemocoel within the first 6 h, with the majority (~90%) in the first 2 h PBM [7, 8]. There was no effect of *CqIR* dsRNA injection on mf ingestion or penetrating the gut epithelium to move to the hemocoel (Fig 2A). However, at 13 days PBM, the *CqIR* dsRNA injected mosquitoes had no infective L3 larvae either in the thorax or the head cavity (Fig 2B). Previous studies found that LF parasites elicit an immune response, e.g., melanotic encapsulation, in the non-vector model species, *Ae*. *aegypti* (Liverpool strain), but go undetected in natural vectors, such as *C*. *quinquefasciatus* [24, 25]. We did not observe worm encapsulation or melanization in the *CqIR* dsRNA injected mosquitoes. The absence of L3 *Wb* in the *CqIR* dsRNA injected mosquitoes may be due to the death of L1 worms prior to reaching or entering in thoracic muscles or failure to develop to the L2 stage because of limited nutrients resulting from decreased trypsin activity during blood digestion. Dead filarial worms are easily overlooked, therefore histochemical stains will be used in follow-up studies to pinpoint exactly where and when *Wb* mortality occurs in *CqIR* dsRNA injected mosquitoes to better understand possible causes.

Our data demonstrated that insulin signaling plays an important role in the activation of trypsin-like enzyme secretion in the midgut and ecdysteroid production by ovaries in *Culex* mosquitoes in a manner similar to that reported for *Ae*. *aegypti*. Ingestion of a blood meal by *Ae*. *aegypti* results in the biphasic release of trypsin-like enzymes by the midgut [26, 27]. Late phase trypsin-like activity occurs between 12 and 30 h PBM [27], and digests most of the blood meal. We previously showed that insulin and Target of Rapamycin (TOR) signaling interact to regulate the timing of late phase trypsin-like gene expression (late trypsin and serine protease VI) and blood meal digestion in *Ae*. *aegypti* [15]. In this study, *Wb* infected *eGFP* control mosquitoes had significantly lower trypsin activity compared to *eGFP* control un-infected mosquitoes, and *CqIR* knockdown and infected females had even lower trypsin activity (Figs 3 and 5). Similarly, *Ae*. *aegypti* infected with *B*. *malayi* had lower transcript levels of four serine proteases and sterol trafficking genes suggesting altered blood digestion/proteolysis in the presence of filarial parasites [8]. We suspect that digestion might be influenced because of increased immune response. When infected with parasite, the mosquito may be allocating more resources towards fighting off the infection instead of blood digestion.

*Aedes* mosquitoes’ flight muscle cells become devoid of glycogen granules following infection with *Brugia* parasites [10, 11] suggesting stored carbohydrates are mobilized during parasite development. Insulin signaling regulates carbohydrate and lipid storage in sugar-fed *Ae*. *aegypti*, as shown by IR knockdown [13]. Although we did not measure the lipid and carbohydrate levels in *C*. *quinquefasciatus* in this study, we noticed that IR knockdown females had less fat deposits than the control (personal observation) and the complete block of parasite development in *CqIR* knockdown females may in part be due to lower nutrient stores available to the developing nematode.

Nutrients are limiting factors for both egg production by mosquito vectors and development of infective pathogens. The total number of eggs laid by female mosquitoes depends upon the mobilization of nutrients stored from the feeding larval stage or previous blood meals and those released by blood digestion [28]. A blood meal also stimulates ovaries to produce ecdysteroids, mediated in part by ILPs [29], and subsequently both amino acids from the blood meal and ecdysteroids activate secretion of vitellogenin and other yolk proteins by the fat body for provisioning eggs to be used by developing embryos. *Brugia* and *Dirofilaria* nematode worms have an adverse effect on mosquito fecundity, with up to a 33% reduction in egg production [30]. A recent study of *Ae*. *aegypti* infected with *B*. *malayi* also supported these earlier findings [31]. Here we found that the ovaries of blood fed, *Wb* infected, *eGFP* dsRNA injected mosquitoes produced similar amounts of ecdysteroids *in vitro* as that of the uninfected controls (Fig 4). In contrast, the ovaries of *CqIR* knockdown females produced significantly lower amounts of ecdysteroids than that of the *eGFP* controls. Therefore, the complete shutdown of egg production in *CqIR* knockdown and infected mosquitoes likely was due to both lower levels of ecdysteroids and nutrients from blood digestion.

Most studies on mosquito-filarial worm interactions are based on the *Ae*. *aegypti- B*. *malayi* system. Although relative ease of use in the laboratory for this system provides advantages, *Ae*. *aegypti* is not a natural vector of filarial worms [32]. The *Ae*. *aegypti* Liverpool strain was genetically selected for susceptibility to many pathogens, including *B*. *malayi*. In contrast, the results of our study were obtained with a native *C*. *quinquefasciatus-Wb* association, thus providing a better assessment of the role of insulin signaling on mosquito physiology and parasite worm development in the field. To further understand the mechanism of *Wb* developmental arrest or mortality in IR knockdown mosquitoes, we will examine the global metabolome and transcriptome in order to pinpoint the metabolites or gene products needed for the parasite to develop.

## References

[1] Budge PJ, Little KM, Mues KE, Kennedy ED, Prakash A, Rout J, et al Impact of Community-Based Lymphedema Management on Perceived Disability among Patients with Lymphatic Filariasis in Orissa State, India. PLoS Neg Trop Dis. 2013; 7(3): e2100 https://doi.org/10.1371/journal.pntd.0002100. 10.1371/journal.pntd.0002100 PMC3597476 23516648

[2] Mutheneni SR, Upadhyayula SM, Kumaraswamy S, Kadiri MR, Nagalla B. Influence of socioeconomic aspects on lymphatic filariasis: A case-control study in Andhra Pradesh, India. J Vector Borne Dis. 2016; 53(3): 272–8. 27681551

[3] Webster JP, Molyneux DH, Hotez PJ, Fenwick A. The contribution of mass drug administration to global health: past, present and future. Philosophical Trans Royal Soc B: Biological Sci. 2014; 369(1645). http://doi.org/10.1098/rstb.2013.0434 10.1098/rstb.2013.0434 PMC4024227 24821920

[4] Molyneux DH, Savioli L, Engels D. Neglected tropical diseases: progress towards addressing the chronic pandemic. Lancet. 2016; 389 (10066): 312–25 doi: 10.1016/S0140-6736(16)30171-4 27639954 10.1016/S0140-6736(16)30171-4

[5] Bockarie MJ, Pedersen EM, White GB, Michael E. Role of Vector Control in the Global Program to Eliminate Lymphatic Filariasis. Ann Review Entomol. 2009; 54: 469–87 10.1146/annurev.ento.54.110807.090626 18798707

[6] Erickson SM, Thomsen EK, Keven JB, Vincent N, Koimbu G, Siba PM et al Mosquito-Parasite Interactions Can Shape Filariasis Transmission Dynamics and Impact Elimination Programs. PLoS Neg Trop Dis. 2013; 7(9): e2433 http://doi.org/10.1371/journal.pntd.0002433 10.1371/journal.pntd.0002433 PMC3772046 24069488

[7] Gad AM, Farid HA, Hammad RE, Hussein MA, Kaschef AH. Host-parasite relationships of Wuchereria bancrofti and mosquito hosts, Culex pipiens L. and Aedes caspius pallas. J Egypt Soc Parasitol. 1996; 26(1): 93–104. 8721232

[8] Michael E, Gambhir M. Transmission models and management of lymphatic filariasis elimination. Adv Exper Med Biol. 2010; 673:157–71. doi: 10.1007/978-1-4419-6064-1_11 20632536 10.1007/978-1-4419-6064-1_11

[9] Erickson SM, Xi Z, Mayhew GF, Ramirez JL, Aliota MT, Christensen BM et al Mosquito Infection Responses to Developing Filarial Worms. PLoS Neg Trop Dis. 2009; 3(10): e529 http://doi.org/10.1371/journal.pntd.0000529 10.1371/journal.pntd.0000529 PMC2752998 19823571

[10] Kan SP, Ho BC. Development of Brugia pahangi in the flight muscles of Aedes togoi: Ultrastructural changes in the infected muscle fibers and the infecting filarial larvae. Am J Trop Med Hyg. 1973; 22: 179–188. 4688414 10.4269/ajtmh.1973.22.179

[11] Lehane MJ, Laurence BR. Flight muscle ultrastructure of susceptible and refractory mosquitoes parasitized by larval Brugia pahangi. Parasitol. 1977; 74: 87–92. 10.1017/s0031182000047557 14324

[12] Marquez AG, Pietri JE, Smithers HM, Nuss A, Antonova Y, Drexler AL et al Insulin-like peptides in the mosquito Anopheles stephensi: identification and expression in response to diet and infection with Plasmodium falciparum. Gen Comp Endocrinol. 2011; 173(2): 303–12. http://doi.org/10.1016/j.ygcen.2011.06.005 21703270 10.1016/j.ygcen.2011.06.005 PMC3153599

[13] Brown MR, Clark KD, Gulia M, Zhao Z, Garczynski SF, Crim JW et al An insulin-like peptide regulates egg maturation and metabolism in the mosquito Aedes aegypti. Proc Natl Acad Sci USA. 2008; 105(15): 5716–5721. http://doi.org/10.1073/pnas.0800478105 18391205 10.1073/pnas.0800478105 PMC2311378

[14] Wen Z, Gulia M, Clark KD, Dhara A, Crim JW, Strand MR et al Two insulin-like peptide family members from the mosquito Aedes aegypti exhibit differential biological and receptor binding activities. Mol Cell Endocrinol. 2010; 328(1–2): 47–55. http://doi.org/10.1016/j.mce.2010.07.003 20643184 10.1016/j.mce.2010.07.003 PMC2957182

[15] Gulia-Nuss M, Robertson AE, Brown MR, Strand MR. Insulin-Like Peptides and the Target of Rapamycin Pathway Coordinately Regulate Blood Digestion and Egg Maturation in the Mosquito Aedes aegypti. PLoS ONE. 2011; 6(5): e20401 http://doi.org/10.1371/journal.pone.0020401 21647424 10.1371/journal.pone.0020401 PMC3103545

[16] Castillo J, Brown MR, Strand MR. Blood Feeding and Insulin-like Peptide 3 Stimulate Proliferation of Hemocytes in the Mosquito Aedes aegypti. PLoS Pathogens. 2011; 7(10): e1002274 http://doi.org/10.1371/journal.ppat.1002274 21998579 10.1371/journal.ppat.1002274 PMC3188524

[17] Zhao YO, Kurscheid S, Zhang Y, Liu L, Zhang L, Loeliger K, Fikrig E. Enhanced Survival of Plasmodium-Infected Mosquitoes during Starvation. PLoS ONE. 2012; 7(7): e40556 http://doi.org/10.1371/journal.pone.0040556 22808193 10.1371/journal.pone.0040556 PMC3393683

[18] Pietri JE, Pietri EJ, Potts R, Riehle M, Luckhart S. Plasmodium falciparum suppresses the host immune response by inducing the synthesis of insulin-like peptides (ILPs) in the mosquito Anopheles stephensi. Develop Comp. 2015; 53(1): 134–44. 10.1016/j.dci.2015.06.012 PMC4536081 26165161

[19] Corby-Harris V, Drexler A, Watkins de Jong L, Antonova Y, Pakpour N, Ziegler R, et al Activation of Akt Signaling Reduces the Prevalence and Intensity of Malaria Parasite Infection and Lifespan in Anopheles stephensi Mosquitoes. PLoS Pathogens. 2010; 6(7): e1001003 http://doi.org/10.1371/journal.ppat.1001003 20664791 10.1371/journal.ppat.1001003 PMC2904800

[20] Singh J. J.S.B. Stain- A review. Indian J Malarial. 1956; 10: 117–12. 13366404

[21] Reuben R, Tewari SC, Hiriyan J, Akiyama J. Illustrated key to genera of Culex (Culex) associated with Japanese encephalitis in Southeast Asia ((Diptera: Culicidae). Mosq Syst. 1994; 26:75–96

[22] Okuda K, Caroci AD, Riobolla PEM, deBianchi AG, Bijovsky AT. Functional morphology of adult female Culex quinquefasciatus midgut during blood digestion. Tissue Cell. 2002;34 (3): 210–19. https://doi.org/10.1016/S0040-8166(02)00032-0 12182814 10.1016/s0040-8166(02)00032-0

[23] Baldridge GD, Feyereisen R. Ecdysteroid titer and oocyte growth in the northern house mosquito, Culex pipiens L. Comp Biochem Physiol- Part A: Physiology. 1986; 83(2): 325–329. doi: 10.1016/0300-9629(86)90583-9 10.1016/0300-9629(86)90583-9 2869872

[24] Beerntsen BT, Luckhart S, Christensen BM. Brugia malayi and Brugia pahangi: inherent difference in immune activation in the mosquitoes Armigeres subalbatus and Aedes aegypti. J Parasitol. 1989;75: 76–81. doi: 10.2307/3282940 2563767

[25] Christensen BM. Immune mechanisms and mosquito-filarial worm relationships, pp. 145–160 in Immune Mechanisms in Znuertehate Vectm, edited by Laciue A. M. 1986 Clarendon Press, Oxford

[26] Lu SJ, Pennington JE, Stonehouse AR, Mobula MM, Wells MA. Reevaluation of the role of early trypsin activity in the transcriptional activation of the late trypsin gene in the mosquito Aedes aegypti. Insect Biochem Mol Biol. 2006;36: 336–343. doi: 10.1016/j.ibmb.2006.01.011 16551547 10.1016/j.ibmb.2006.01.011

[27] Noriega FG, Colonna AE, Wells MA. Increase in the size of the amino acid pool is sufficient to activate translation of early trypsin mRNA in Aedes aegypti midgut. Insect Biochem Mol Biol. 1999; 29: 243–247. 10319437 10.1016/s0965-1748(98)00132-5

[28] Van Handel E. Metabolism of nutrients in the adult mosquito. Mosquito News. 1984;44(4): 573–79

[29] Nuss AB, Brown MR. Isolation of an insulin-like peptide from the Asian malaria mosquito, Anopheles stephensi, that acts as a steroidogenic gonadotropin across diverse mosquito taxa. Gen Comp Endocrinol. 2018; 258:140–48. http://dx.doi.org/10.1016/j.ygcen.2017.05.007 28502740 10.1016/j.ygcen.2017.05.007 PMC5681901

[30] Hurd H. Parasite manipulation: stretching the concepts. Beh Processes. 2005; 68(3): 235–236. http://doi.org/10.1016/j.beproc.2004.07.010 10.1016/j.beproc.2004.07.010 PMC2474665 15792696

[31] Gleave K, Cook D, Taylor MJ, Reimer LJ. Filarial infection influences mosquito behaviour and fecundity. Sci Rep. 2016; 6: 36319 http://doi.org/10.1038/srep36319 27796352 10.1038/srep36319 PMC5087081

[32] Lindsay SW, Denham DA. The ability of Aedes aegypti mosquitoes to survive and transmit infective larvae of Brugia pahangi over successive blood meals. J Helminthol. 1986;60: 159–68. 3745870 10.1017/s0022149x00026031

